# ZnS Quantum Dot Based Acetone Sensor for Monitoring Health-Hazardous Gases in Indoor/Outdoor Environment

**DOI:** 10.3390/mi12060598

**Published:** 2021-05-22

**Authors:** Rajneesh Kumar Mishra, Gyu-Jin Choi, Hyeon-Jong Choi, Jin-Seog Gwag

**Affiliations:** Department of Physics, Yeungnam University, Gyeongsan 38541, Gyeongbuk, Korea; gyujin1125@gmail.com (G.-J.C.); dkssud229@naver.com (H.-J.C.)

**Keywords:** ZnS quantum dots (QDs), acetone gas sensing, selectivity, stability, quick response and recovery time, gas sensing mechanisms

## Abstract

This study reports the ZnS quantum dots (QDs) synthesis by a hot-injection method for acetone gas sensing applications. The prepared ZnS QDs were characterized by X-ray diffraction (XRD) and transmission electron microscopy analysis. The XRD result confirms the successful formation of the wurtzite phase of ZnS, with a size of ~5 nm. Transmission electron microscopy (TEM), high-resolution TEM (HRTEM), and fast Fourier transform (FFT) images reveal the synthesis of agglomerated ZnS QDs with different sizes, with lattice spacing (0.31 nm) corresponding to (111) lattice plane. The ZnS QDs sensor reveals a high sensitivity (92.4%) and fast response and recovery time (5.5 s and 6.7 s, respectively) for 100 ppm acetone at 175 °C. In addition, the ZnS QDs sensor elucidates high acetone selectivity of 91.1% as compared with other intrusive gases such as ammonia (16.0%), toluene (21.1%), ethanol (26.3%), butanol (11.2%), formaldehyde (9.6%), isopropanol (22.3%), and benzene (18.7%) for 100 ppm acetone concentration at 175 °C. Furthermore, it depicts outstanding stability (89.1%) during thirty days, with five day intervals, for 100 ppm at an operating temperature of 175 °C. In addition, the ZnS QDs acetone sensor elucidates a theoretical detection limit of ~1.2 ppm at 175 °C. Therefore, ZnS QDs can be a promising and quick traceable sensor nanomaterial for acetone sensing applications.

## 1. Introduction

The sensing of volatile organic compounds (VOCs) has attracted much attention due to its production in the chemical industry and research laboratory and its effects on humans [1,2,3,4]. Chemical gas sensors have received much consideration in the real-time sensing of health-hazardous gases, which play a dynamic role in industrial and research settings and disease diagnosis [5,6]. The resistive gas sensor is the most significant among various gas sensors due to its simple configuration, easy fabrication, and low cost. The gas sensing mechanism of the inorganic chemo-resistive gas sensor depends on the change in resistance of a sensing element during target gas sensing [7].

Among various VOCs, acetone is highly dangerous for human health [8]. The inhalation of an excess amount of acetone can lead to several health complications, such as fatigue, cardiovascular disease, headache, respiratory disease, lung cancer, and can even be harmful to the human nervous system [8,9,10]. Acetone is colorless and transparent and can be seen to be more promising during the exhaled breath due to its non-invasive nature [11]. The acetone level increased in the human body by 2 to 5 ppm when body fat decomposition rates reached 2 g h^−1^ [12]. The healthy human body contains an acetone concentration of 0.3–0.9 ppm; however, exhaled acetone concentration reaches 1.8 ppm by carbohydrate digestion, and normal glucose metabolism causes a severe risk of type 1 diabetes [13,14]. Human breathing analysis has recently included a rapid and non-invasive diagnostic technique for recognizing acetone in exhaled human breath [15]. The exhaled breath inspection is an evolving technology in medical diagnostics and has the advantages of being low-cost and non-invasive. Comprehensive research has been dedicated to the progress of gas sensing technology; therefore, gas chromatography-mass spectroscopy (GC-MS) has been employed in clinical trials to diagnose diabetes, renal failure, and lung cancer [16,17]. However, this technology needs high-priced, heavy equipment and well-trained personnel. Therefore, it is highly viable to diagnose diabetes non-invasively through exhaled human breath inspection. The exhaled breath sensors based on nanomaterials have not been demonstrated commercially for clinical diagnostic tools due to their out-of-range exhaled acetone detection limit of 50–500 ppm [18,19]. Therefore, the development of inexpensive, high-performance, stable, selective, and high-precision acetone sensors for clean, green, and safe working places is paramount [18,19].

The selection of suitable gas sensing element material is crucial for enhancing the sensing properties of the gas sensor. Among various transition metal chalcogenides, zinc sulfide (ZnS), a significant II-IV compound semiconductor, has been extensively investigated due to its excellent electrical, optical, and catalytic properties [20]. It has a wide optical direct bandgap of 3.7 eV and exciton binding energy of 40 meV, which offers both n- and p-type dopings [21]. ZnS has fertile interstitial sulfur lattice defects, trapped surface states, and sulfur vacancies, enhancing surface oxygen adsorption, which further facilitates enough surface-adsorbed o^-^ ion species [22,23,24]. These adsorbed ion species boost interactions between the sensing materials and gas molecules, which leads to the high gas sensing performance of the ZnS sensor. The various types of ZnS nanostructures, such as nanotubes [25], quantum dots [26], nanoribbons [27], nanowires [28], etc., have been synthesized and expansively studied for various potential applications. In addition, numerous efforts have been made to explore their applications in different devices, such as solar cells [29], light-emitting diodes [30], ultraviolet laser [31], batteries [32], supercapacitors [33], and gas sensors [34].

Several literature reports have focused on the transition metal dichalcogenides sensing element-based gas sensors for acetone sensing applications. Chang et al. reported on the ZnO/MoS_2_ based acetone sensor [35]. The sensor showed the highest response of 4.67 for 20 ppm concentration at 100 °C under UV light. It was also observed that the sensor shows quick response (56 s) and recovery time (69 s) for 20 ppm concentration at 100 °C under UV light. Jahromi et al. studied the lead sulfide nanosheet-based acetone sensor [36]. The sensor showed the highest response of 24.16% for 1 ppm concentration at an operating temperature of 25 °C. Ma et al. investigated the SnS/rGO gas sensing element for acetone sensor applications [37]. The prepared sensor represented the highest response of 20.2 for 25 ppm concentration acetone at 100 °C. However, the high response and recovery time and poor stability of the gas sensor demand researchers’ urgent attention, to investigate for quick detection as well as selective and highly stable gas sensing performance.

This study reports acetone gas sensing properties based on ZnS QDs as a sensing element synthesized by the hot-injection method. The ZnS QDs sensor displays fast response and recovery time. In addition, it shows high sensitivity, nailing selectivity, and superior stability for 100 ppm acetone concentration at an operating temperature of 175 °C. In addition, Table S1 presents the comparison of the ZnS nanostructures acetone sensor with other similar reports or research devices based on metal-oxide nanomaterials. Therefore, the ZnS QDs sensor can be a promising sensor for detecting acetone vapor from the exhaled breath for the invasive type 1 diabetes inspection. We also explored the gas sensing mechanisms, schematic visualizations of the formation of the depletion region on the ZnS QDs, and possible reactions with acetone molecules.

## 2. Materials and Methods

### 2.1. Materials Synthesis

The zinc acetate hydrate, oleylamine, thioacetamide, ethanol, polyvinylidene fluoride (PVDF), and N-Methyl-2-pyrrolidone (NMP) were purchased from Sigma-Aldrich and used as received.

The ZnS quantum dots (QDs) were synthesized by the hot-injection method, as discussed below. In a typical synthesis procedure, 0.5 mmol of zinc acetate hydrate and 30 mL of oleylamine were mixed in a 50 mL three-neck flask using magnetic stirring under the heater mantle. The zinc acetate hydrate/oleylamine mixture was degassed and filled with N_2_ two times at room temperature and then heated to 110 °C for 30 min using the heater mantle. Further, the zinc acetate hydrate/oleylamine mixture’s temperature was raised to 160 °C, and 2 mL of sulfur (S) source solution was injected. S solution was prepared by mixing 0.1 M of thioacetamide into 30 mL of oleylamine. Then, the temperature was set at 120 °C and degassed for 10 min; after this, it was raised to 230 °C in 5 min for 20 min, and the reaction was quenched by removing it from the heater mantle. Finally, the ZnS QDs were washed and collected by centrifugation and dried under vacuum at 75 °C for 10 h.

### 2.2. Material Characterizations

X-ray diffraction (XRD) (PANalytical with Cu Kα radiation, X’Pert PRO, Almelo, Netherlands) was utilized to investigate the ZnS QDs crystal structure. Transmission electron microscopy (TEM) (JEOL JEM 2100F, JEOL Ltd., Tokyo, Japan) was used to study morphology, particle size, lattice spacing, and crystal phase of ZnS QDs.

### 2.3. ZnS Quantum Dots (QDs) Based Gas Sensor Fabrication and Measurements

The acetone gas sensing properties of the ZnS QDs based sensor were investigated at various concentrations (20–100 ppm) at different operating temperatures (100–200 °C). For the film deposition, we prepared a solution of ZnS QDs, using a polyvinylidene fluoride (PVDF) binder (9 mg ZnS QDs and 1 mg PVDF) and N-Methyl-2-pyrrolidone (NMP) as solvent. The ZnS QDs based gas sensor was fabricated using a spin coating process on a glass substrate (accelerating time 10 s at 1000 rpm; spin coating at 2000 rpm for 30 s; de-accelerating time 10 s at 1000 rpm). After spin coating, the film was dried in a hot air oven at 70 °C for 1 h, and the spin coating process was repeated. Further, we repeated the spin coating and drying process 25 times to make a uniform film. After this, silver paint was applied using a paintbrush to coat the conducting electrodes (for making electrical contacts) (Figure S1). During the gas sensing study, the gas sensor was allowed to calibrate for 25 min in the gas sensing chamber until sensor resistance became nearly constant. The temperature inside the gas chamber was observed using a Motwane-454 digital multimeter. The sensor resistance was investigated using a Keithley- 2100 digital multimeter for various acetone concentrations (using Hamilton micro-syringe) and operating temperatures. The schematic representation of static gas sensing setup is shown in Figure S2. The atmospheric humidity was nearly 23 R.H. (±4) during gas sensing tests. The required amount of liquid acetone concentration was calculated by the following Equation (1) [38]:

(1) $$C=\frac{22.4\times \varphi \times \rho \times V_{1}}{M\times V_{2}}\times 1000\;\mathrm{ppm}$$

where *C* is the required acetone concentration, φ is the acetone gas volume fraction, ρ (g mL^−1^) is the density of the liquid acetone, $V_{1}\;$(μL) and $V_{2}\;$(L) are the volumes of the acetone and gas chamber, respectively, and M (g mL^−1^) is the molecular weight of the liquid acetone.

## 3. Results and Discussion

### 3.1. Structural and Morphological Studies

**Scheme 1:**
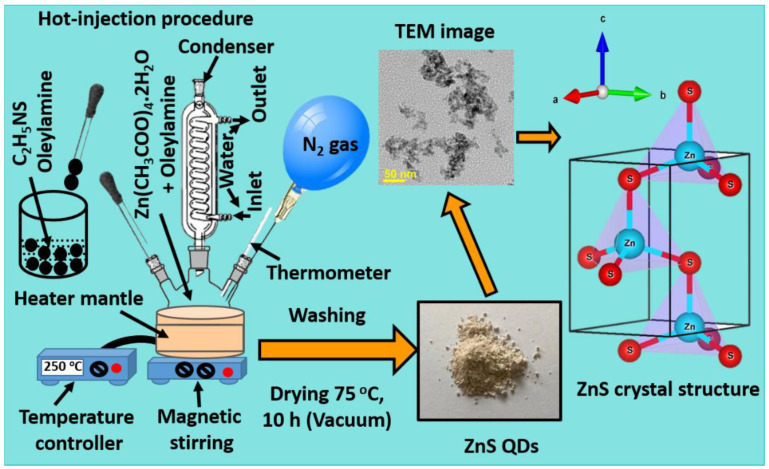
Schematic representation of the ZnS quantum dots (QDs) using the hot-injection method and illustration of the crystal structure of the ZnS.

Scheme 1 shows the preparation of the ZnS QDs using the hot-injection method and a visualization of the crystal structure. The detailed preparation procedure as shown in Scheme 1 is discussed in the material synthesis section.

**Figure 1:**
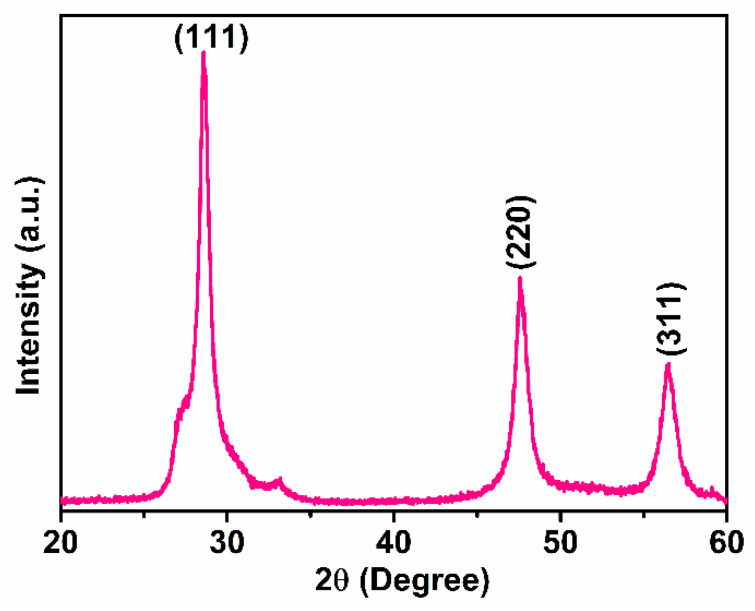
X-ray diffraction (XRD) spectrum of the as-prepared ZnS quantum dots.

The structural properties of the as-prepared ZnS quantum dots (QDs) were studied using XRD. Figure 1 shows the X-ray diffraction (XRD) spectrum of ZnS QDs, with three intense peaks positioned at 28.63°, 47.58°, and 56.52° corresponding to the lattice spacing 0.31 nm, 0.19 nm, and 0.16 nm, which represents (111), (220), and (311) lattice planes, respectively. The XRD peaks of the ZnS QDs are consistent with the wurtzite structure of ZnS (JCPDS card No. 05-0566) [39]. It is observed that the synthesized material is composed of ZnS, which further justifies the high purity synthesis. The crystallite size (*CS*) and lattice strain (*LS*) of the ZnS QDs were evaluated using the Debye-Scherrer formula and tangent formula Equations (2) and (3), respectively [40].

(2) $$CS=\frac{0.9\lambda }{\beta \cos\theta }$$

(3) $$LS=\frac{\beta }{4\tan\theta }$$

where *λ* is the X-ray wavelength (1.54056 Å), *θ* is the Braggs diffraction angle, and *β* (radians) is the full width at half-maximum of diffractions peaks. The average crystallite size and lattice strain are 9.8 nm and 0.0099, respectively.

**Figure 2:**
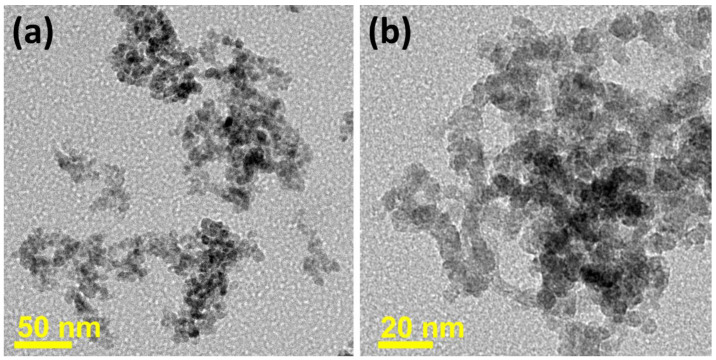
Transmission electron microscopy images of ZnS QDs at different magnifications. (**a**) Agglomerated and overlapped QDs, and (**b**) high magnification image illustrating the different sizes QDs.

Figure 2a,b illustrates the TEM images of ZnS QDs at different magnifications. The nucleation and growth of ZnS quantum dots can be described as the dissolution and crystallization mechanism, in which the Zn and S precursor particles dissolve in the solution during stirring and heating in the three-neck flask during the hot-injection method. TEM images are composed of interconnected, agglomerated, and overlapped quantum dot structures of ZnS, having the size of nearly 5 nm. It is also demonstrated that the as-prepared quantum dots keep a high surface-to-volume ratio due to their zero-dimensional nature, which ensures fast chemisorption, excellent conductivity, and good conduction during gas sensing measurements. The different sizes of QDs can also behave like electron transporters during gas sensing analysis.

**Figure 3:**
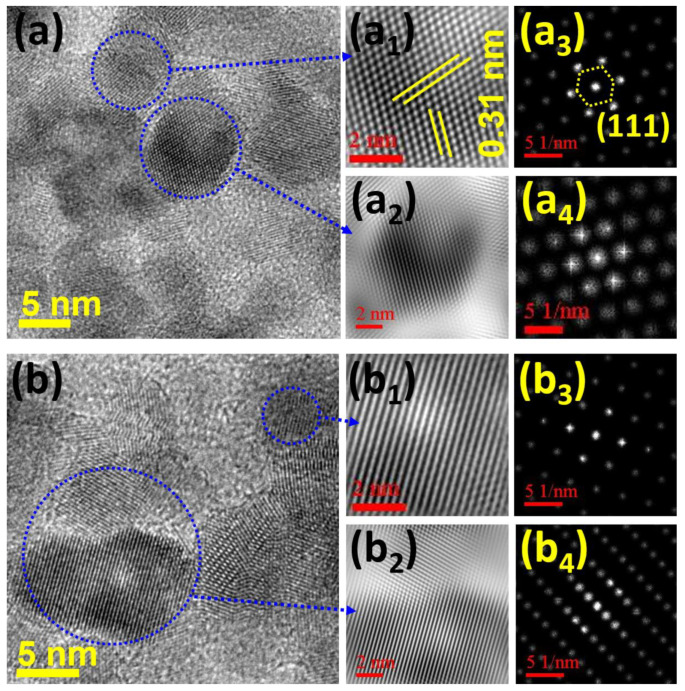
(**a**,**b**) High-resolution transmission electron microscopy (HRTEM) images: (**a_1_**,**a_2_**) and (**b_1_**,**b_2_**) specific area HRTEM images and their corresponding (**a_3_**,**a_4_**) and (**b_3_**,**b_4_**) FFT patterns of the ZnS QDs.

Figure 3a,b shows the high-resolution transmission electron microscopy (HRTEM) images of the as-prepared ZnS QDs. It is seen that there are many different sizes of ZnS QDs, with 2 to 10 nm. Figure 3(a_1_,a_2_) illustrates the HRTEM images of ZnS QDs from the zoomed portion of Figure 3a. It is realized that the two QDs are overlapped and cross-connected with each other, corresponding to the lattice spacings of 3.1 nm. Figure 3(a_3_,a_4_) depicts the fast Fourier transform spectroscopy (FFT) patterns of the selected area, as shown in Figure 3(a_1_,a_2_), of the ZnS QDs. FFT patterns in Figure 3(a_3_,a_4_) show the lattice planes of (111) corresponding to the lattice spacing of 0.31 nm. Figure 3(b_1_,b_2_) elucidates the HRTEM images of ZnS QDs from the enlarged portion of Figure 3b. It is recognized that the single QDs (Figure 3(b_1_)) and three QDs (Figure 3(b_2_)) are observed and interconnected with each other, which is consistent with the lattice spacings of 3.1 nm. Figure 3(b_3_,b_4_) portrays the FFT patterns of the designated area, as shown in Figure 3(b_1_,b_2_), of the ZnS QDs. FFT patterns in Figure 3(b_3_,b_4_) display the lattice planes of (111) corresponding to the lattice spacing of 0.31 nm, which is the most intense peak observed in the XRD spectrum (Figure 1). The TEM and HRTEM results conclude the successful formation of the ZnS QDs structure with the wurtzite phase, justifying the XRD results.

### 3.2. Acetone Gas Sensing Studies

The gas sensitivity *S*(%) of the ZnS QDs acetone sensor is evaluated by the following Equation (4) [41]:

(4) $$S\left(\%\right)=\frac{R_{a}-R_{g}\;}{R_{a}}\times 100$$

where *R_a_* and *R_g_* are the ZnS QDs acetone sensor resistance in air and gas, respectively.

**Figure 4:**
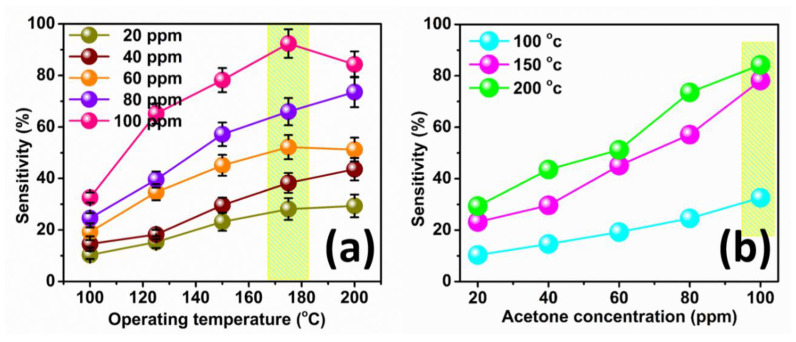
(**a**) Sensitivity vs. operating temperature plots and (**b**) sensitivity vs. concentration plots of ZnS QDs based acetone sensor.

Figure 4a,b shows the ZnS QDs sensor’s acetone sensing properties for various concentrations (20–100 ppm) at different operating temperatures (100–200 °C). Figure 4a shows the sensitivity plots for acetone gas of the ZnS QDs sensor. The ZnS QDs sensor was also tested for low acetone concentrations of 10 and 15 ppm. The sensor did not show any response for 10 ppm; however, it showed a nearly negligible sensitivity of 0.05% for 15 ppm at 175 °C. It is seen that the sensitivity of the ZnS QDs sensor increases with operating temperature and gas concentration. It indicates the highest acetone sensitivity of 92.4% for 100 ppm concentration at 175 °C. The following are possible reasons for fast and high acetone sensitivity: (i) quantum dots with high surface area provide large chemisorption of acetone molecules, which results in high sensitivity, and (ii) QDs can also offer fast adsorption/desorption of oxygen species and their quick interaction with acetone molecules due to the quantum size, resulting fast response and recovery time [42,43]. However, it is also observed that the sensitivity decreases with an operating temperature of 180 °C. This may be due to various factors: (i) more adsorption of atmospheric oxygen O_2_ and its conversion into active site O^−^ can deplete the conduction path, which causes enhanced potential barrier height at the grain boundaries, (ii) unavailability of active sites O^−^, and (iii) the quick vaporization of acetone molecules and their cloud formation around the sensor surface at ambient temperature causes the slow sensitivity [44,45,46,47]. Figure 4b displays the sensitivity plots of acetone sensing properties of the ZnS QDs sensor for all concentrations at three different working temperatures (100 °C, 150 °C, 200 °C). The sensitivity increases with operating temperature and acetone concentration. The ZnS QDs sensor follows the linear relationship between the sensitivity and acetone concentration. The acetone gas response depends on the balance between the rate of acetone molecule diffusion and oxidation reaction. In addition, the chemisorption, adsorption, and desorption rate of acetone molecules and active sites (O^−^) on the surface of the ZnS QDs sensor and the carrier concentration of the gas sensing body also play a crucial role in the acetone sensitivity [5,6,7,8]. At low operating temperature (below 140 °C), the ZnS QDs sensor’s acetone sensitivity is low due to insufficient activation energy. However, at high operating temperature (above 140 °C), the ZnS QDs sensor’s acetone sensitivity is high because of high thermal activation energy [5,6,7,8].

**Figure 5:**
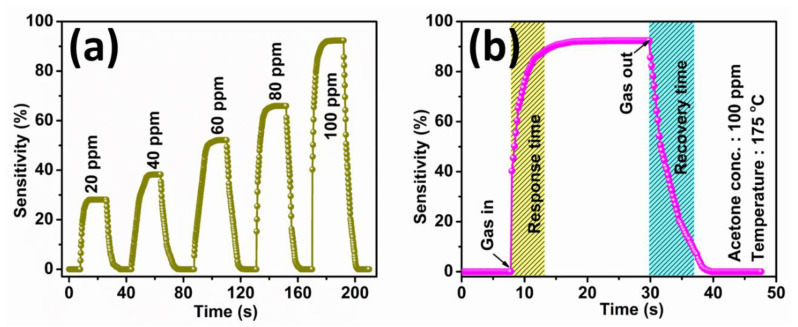
(**a**) Transient characteristics at 175 °C and (**b**) response and recovery time calculation of ZnS QDs based acetone sensor.

Figure 5a conveys the transient response and recovery time characteristics of the ZnS QDs sensor for various acetone concentrations at 175 °C. The sensor response steadily increased and found the highest response at 175 °C during acetone concentration raised from 20 ppm to 100 ppm, enhancing the chemisorption rate of acetone molecules on the sensor surface. Figure 5b depicts the calculation of response time and recovery time when the ZnS QDs sensor reaches and recovers 90% of its maximum and initial value, respectively. It is observed that the ZnS QDs sensor shows a quick response time (5.5 s) and speedy recovery time (6.7 s) towards 100 ppm acetone at 175 °C. This may be due to the high surface area, activation energy, low potential barrier, fast adsorption, and desorption rate on the surface of the ZnS QDs. The theoretical detection limit of the ZnS QDs acetone sensor was calculated as shown in Figure S3 and Equations (S1) and (S2). Therefore, the estimated theoretical detection limit of the ZnS QDs acetone sensor is ~1.2 ppm at 175 °C.

**Figure 6:**
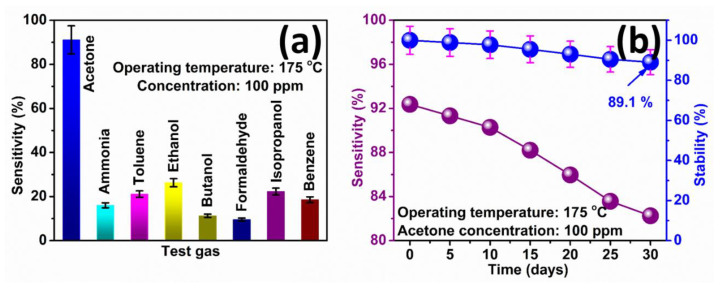
(**a**) Selectivity and (**b**) stability plots of ZnS QDs based acetone sensor.

Figure 6a shows the selectivity of the ZnS QDs acetone sensor in the presence of ammonia, toluene, ethanol, butanol, formaldehyde, isopropanol, and benzene for 100 ppm concentration at an optimum operating temperature of 175 °C. It is observed that the sensor shows the highest sensitivity towards acetone (91.1%) compared with ammonia (16.0%), toluene (21.1%), ethanol (26.3%), butanol (11.2%), formaldehyde (9.6%), isopropanol (22.3%), and benzene (18.7%).

Furthermore, we assessed the selectivity coefficient (*C*) to quantify the acetone selectivity with respect to other gases, using the relation *C* = *S*_acetone_/*S*_othergas_ [44,45]. The selectivity coefficient values of the ZnS QDs acetone sensor are 5.7 (ammonia), 4.3 (toluene), 3.5 (ethanol), 8.1 (butanol), 9.5 (formaldehyde), 4.1 (isopropanol), and 4.9 (benzene). This shows that the selectivity of the ZnS QDs acetone sensor is highest, at 9.5 times higher than formaldehyde and 3.5 times higher than the ethanol, for 100 ppm concentration at 175 °C. Figure 6b elucidates the excellent stability of 89.1% of the ZnS QDs based acetone sensor. It is also observed that the sensitivity decreases from 92.4% to 82.3% during the 30 days of the stability test. The excellent stability of the ZnS QDs sensor may be due to the following reasons: (i) high surface area of quantum dots, leading to more adsorption of ionic species, and (ii) high conductivity of ZnS QDs, leading to quick and sufficient electron transport to interact with the acetone molecules. Table S1 presents a comparison of the ZnS nanostructures acetone sensor with other reports based on metal-oxide nanomaterials published in the literature. It is clearly indicated from Table S1 that the present ZnS QDs acetone sensor illustrates high sensitivity, low theoretical detection limit, quick response and recovery time, and high selectivity and sensitivity.

### 3.3. Gas Sensing Mechanisms

The acetone gas sensing mechanism of the ZnS QDs sensor is typically based on the sensor element’s resistance fluctuation during the gas detection. It essentially depends on the chemisorption reactions between acetone molecules and adsorbed active ions on the sensor surface, which cause a change in the charge depletion layer and regulate the change in resistance [46,47]. The interaction of oxygen molecules with the ZnS QDs sensor surface and ionic active site formation are summarized in Equations (5)–(7) [46,47,48,49].

(5) $$O_{2}\left(gas\right)↔O_{2}\left(ads\right)$$

(6) $$O_{2}\left(ads\right)+e^{-}↔O_{2}^{-}\left(ads\right)$$

(7) $$O_{2}^{-}\left(ads\right)+e^{-}↔2O^{-}\left(ads\right)$$

The anticipated reaction mechanism of the acetone molecules by the adsorbed oxygen active species is addressed in Equation (8) [50].

(8) $$CH_{3}COCH_{3}+8O^{-}\;↔3CO_{2}+3H_{2}O+8e^{-}$$

where the acetone molecules interact with the oxygen active site present on the sensor surface, liberate carbon dioxide and water molecules, and release the electrons to the ZnS QDs conduction band.

**Figure 7:**
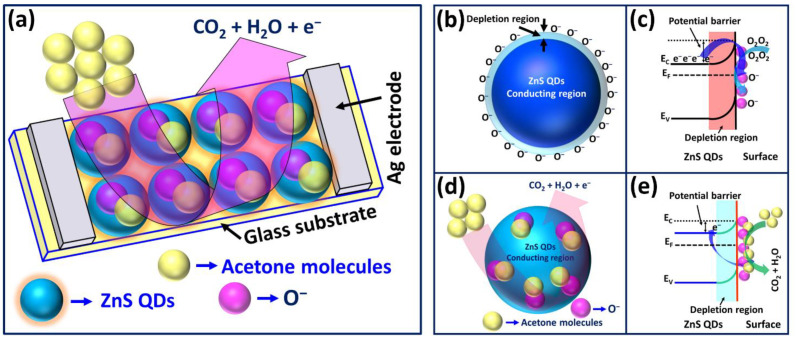
(**a**) Schematic view of ZnS QDs sensor, oxygen adsorption, and acetone reaction; (**b**,**c**) concept of interactions of oxygen molecules and formation of the depletion region on ZnS QDs sensor surface; (**d**,**e**) schematic view of oxygen adsorption, ionic site formation, and chemisorption of acetone molecules on the surface of ZnS QDs.

Figure 7a shows the schematic representation of the fabricated ZnS QDs acetone gas sensor on the glass substrate, the adsorption of oxygen, and the interaction between active sites (O^−^) and acetone molecules. When the sensor is exposed in the gas chamber at ambient temperature, atmospheric oxygen is adsorbed on the ZnS QDs conducting surface and active oxygen sites (O^−^) are formed (Figure 7b), in accordance Equations (5)–(7), and a depletion region is developed. Figure 7c illustrates an electronic band structure of adsorption of oxygen as well as the formation of the potential barrier and depletion region. Figure 7d shows the interaction of the gas molecules with adsorption of atmospheric oxygen on the ZnS QDs conducting surface when the acetone gas is injected into the gas chamber at ambient temperature; this results in the release of carbon dioxide and water molecules and liberates electrons into the conduction band of the ZnS QDs (follows the procedure discussed in Equation (8)). Figure 7e elucidates an electronic band structure of the chemisorption process during acetone molecule sensing and reducing of the potential barrier height and depletion region due to the liberation of more electrons into the conduction band of the ZnS QDs (follows the procedure discussed in Equation (8)).

## 4. Conclusions

In conclusion, the ZnS QDs were successfully synthesized by a hot-injection method for acetone gas sensing applications. The XRD confirms the crystallize size, lattice parameters, lattice strain, and crystal structure of the prepared ZnS QDs. Furthermore, TEM, HRTEM, and FFT results show the size, lattice spacing, lattice planes, and crystal structure of the ZnS QDs, verifying the XRD results. The ZnS QDs sensor shows excellent sensitivity of 92.4% for a 100 ppm concentration of acetone at an operating temperature of 175 °C. The ZnS QDs sensor shows quick response and recovery time for a 100 ppm concentration of acetone at an operating temperature of 175 °C. The ZnS QDs sensor reveals the highest stability of 89.1% during 30 days of its operation, with an interval of 5 days, for 100 ppm at an operating temperature of 175 °C. The ZnS QDs sensor divulges a high selectivity of 91.1% toward acetone compared with other gases for 100 ppm at 175 °C. In addition, we fabricated various devices (ZnS QDs sensor) by trial and error and investigated the optimized device for acetone gas sensing studies. However, the effects of humidity and pressure need to be studied separately in the future. Therefore, ZnS QDs could be an alternative nanomaterial as an acetone sensing element for human breath analysis.

## Supplementary Materials

The following are available online at https://www.mdpi.com/article/10.3390/mi12060598/s1. Figure S1: Digital image of the ZnS QDs sensor; Figure S2: Schematic view of static gas sensing setup; Figure S3: Response vs. concentration plot of the ZnS QDs acetone sensor. Table S1: Comparative study of the acetone gas sensor based on ZnS nanostructures and metal-oxide nanomaterials.
