# Supplementary material for: ZnS Quantum Dot Based Acetone Sensor for Monitoring Health-Hazardous Gases in Indoor/Outdoor Environment
Source: Micromachines (Basel). 2021 May 22;12(6):598. doi: 10.3390/mi12060598 (PMC8224612; doi:10.3390/mi12060598)
Supplement: Supplementary file 1 [file micromachines-12-00598-s001.zip › micromachines-1214430-supplementary.pdf]

micromachines

Supplementary Materials

ZnS Quantum Dot Based Acetone Sensor for Monitoring Health-Hazardous Gases in Indoor/Outdoor Environment

Rajneesh Kumar Mishra, Gyu-Jin Choi, Hyeon-Jong Choi and Jin-Seog Gwag

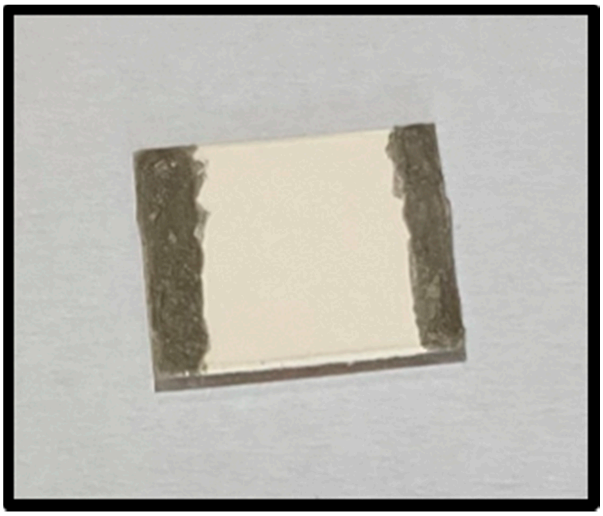

Figure S1. Digital image of the ZnS QDs sensor.

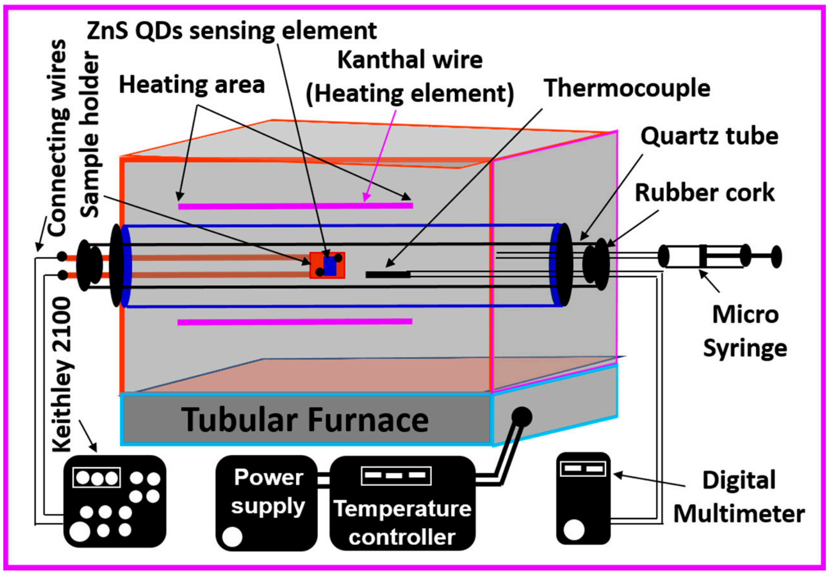

Figure S2. Schematic view of static gas sensing setup.

The theoretical detection limit of the ZnS QDs acetone sensor is calculated at 175 °C using the following relation Equation S1 [1];

$$DL = 3\frac{rms_{noise}}{k} \quad \text{(S1)}$$

Copyright: © 2021 by the authors. Licensee MDPI, Basel, Switzerland. This article is an open access article distributed under the terms and conditions of the Creative Commons Attribution (CC BY) license (http://creativecommons.org/licenses/by/4.0/).

where k is the slope of logarithm plot [Fig. S3], and rms_{noise} is the root-mean-square deviation on the baseline, as discussed in Equation S2;

$$rms_{noise} = \sqrt{\frac{\Sigma(y - y_i)^2}{N}} \tag{S2}$$

where y is the baseline data points, y_i is the average of baseline data points, and N is the number of data points (here, we took five data points).

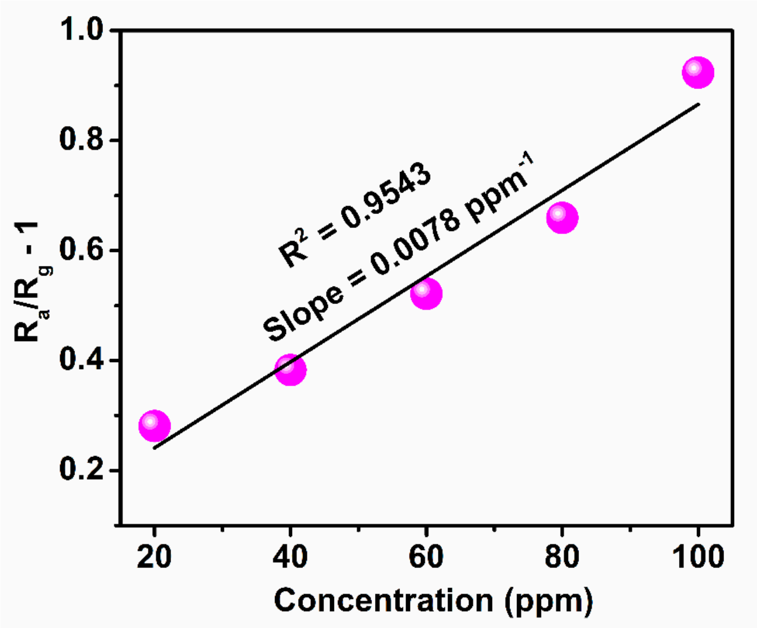

Figure S3. Response vs. concentration plot of the ZnS QDs acetone sensor.

Table S1. Comparative study of the acetone gas sensor based on ZnS nanostructures and metal-oxide nanomaterials.

Sensing Materials	Operating Temperature (°C)	Acetone (ppm)	Sensitivity	Response Time	Recovery Time	Detection limit	Stability / Selectivity	Ref.
ZnS QDs	175	100	92.4 %	5.5 s	6.7 s	1.2 ppm	89.1% (30 days) / 91.1 %	This work
ZnS nanowires	320	100	21.2	~ 30 s	~ 45 s	--	-- / 21.1	2
Au-ZnS	260	100	84.4 %	--	--	--	-- / --	3
ZnO@ZnS core/shell	300	500	~ 9.6	~ 10 s	~ 16 s	--	Cycling for 550 s /-	4
Cr_2O_3-ZnS	300	200	~ 11	--	--	--	-- / --	5
20Nb-WO_3	325	1	~ 16	~ 7 s	~ 625 s	--	~ 14.3 (18 days) / 22.3	6
TiO_2/α-Fe_2O_3	300	300	~ 34	~ 22 s	~ 86 s	--	~ 23 (30 days) / 23	7
NiO/ Zn_2SnO_4	300	100	~ 50	~ 1 s	~ 60 s	< 100 ppb	~ 5 cycles for 1000 s / 49.6	8
Co_3O_4	200	100	~ 8.61	~ 43 s	~ 92 s	--	~ 8.5 (10 days) / 8.61	9
SnO_2/$ZnSnO_3$	290	300	~ 53	~ 5 s	~ 115 s	--	~ 31 (30 days) / 32	10

References:

1. Song, Y.G.; Jung, I.; Shin, J.; Shim, Y.-S.; Kim, G.S.; Ju, B.-K.; Kang, C.-Y. Ionic-activated semiconducting gas sensors operated by piezoelectric generators at room temperature. *Sens. Actuators B* **2021**, *332*, 129481.
2. Wang, X.; Xie, Z.; Huang, H.; Liu, Z.; Chen, D.; Shen, G. Gas sensors, thermistor and photodetector based on ZnS nanowires. *J. Mater. Chem.* **2012**, *22*, 6845.
3. Zhang, L.; Dong, R.; Zhu, Z.; Wang, S. Au nanoparticles decorated ZnS hollow spheres for highly improved gas sensor performances. *Sens. Actuators B* **2017**, *245*, 112–121.
4. Zhang, W.; Wang, S.; Wang, Y.; Zhu, Z.; Gao, X.; Yang, J.; Zhang, H.X. ZnO@ZnS core/shell microrods with enhanced gas sensing properties. *RSC Adv.* **2015**, *5*, 2620–2629.
5. Sun, G.-J.; Kheel, H.; Ko, T.-G.; Lee, C. Prominent Ethanol Sensing with Cr_2O_3 Nanoparticle-Decorated ZnS Nanorods Sensors. *J. Korean Phys. Society* **2016**, *69*, 390–397.
6. Choi, H. J.; Chung, J.-H.; Yoon, J.-W.; Lee, J.-H. Highly selective, sensitive, and rapidly responding acetone sensor using ferroelectric ε-WO_3 spheres doped with Nb for monitoring ketogenic diet efficiency. *Sens. Actuators B* **2021**, 338, 129823.
7. Wang, C.; Wang, Y.; Cheng, P.; Xu, L.; Dang, F.; Wang, T.; Lei, Z. In-situ generated TiO_2/α-Fe_2O_3 heterojunction arrays for batch manufacturing of conductometric acetone gas sensors. *Sens. Actuators B* **2021**, 340, 129926.
8. Zhou, C.; Meng, F.; Chen, K.; Yang, X.; Wang, T.; Sun, P.; Liu, F.; Yan, X.; Shimanoe, K.; Lu, G. High sensitivity and low detection limit of acetone sensor based on NiO/ Zn_2SnO_4 p-n heterojunction octahedrons. *Sens. Actuators B* **2021**, 339, 129912.
9. Fan, X.; Xu, Y.; Ma, C.; He, W. In-situ growth of Co_3O_4 nanoparticles based on electrospray for an acetone gas sensor. *J. Alloys Comp.* **2021**, 854, 157234.
10. Cheng, P.; Lv, L.; Wang, Y.; Zhang, B.; Zhang, Y.; Zhang, Y.; Lei, Z.; Xu, L. SnO_2/$ZnSnO_3$ double-shelled hollow microspheres based high-performance acetone gas sensor. *Sens. Actuators B* **2021**, 332, 129212.
